# Cross-species transcriptomic plasticity and root architectural adaptations in Brachypodium (*Brachypodium distachyon*) and barley (*Hordeum vulgare*) genotypes under different ammonium and nitrate availability

**DOI:** 10.3389/fpls.2026.1916213

**Published:** 2026-10-09

**Authors:** Hamid Rouina, Dilkaran Singh, Christopher Arlt, Amy Marshall-Colon, Benjamin Stich

**Affiliations:** 1 Forschungszentrum Jülich GmbH, Institute of Bio- and Geosciences, IBG-2: Plant Sciences, Jülich, Germany; 2 Faculty of Agriculture, University of Bonn, Bonn, Germany; 3 Department of Plant Biology, University of Illinois at Urbana-Champaign, Urbana-Champaign, IL, United States; 4 Julius Kühn Institute, Federal Research Centre on Cultivated Plants, Institute for Breeding Research on Agricultural Crops, Sanitz, Germany; 5 Faculty of Agricultural and Environmental Sciences, University of Rostock, Rostock, Germany

**Keywords:** barley, *Brachypodium distachyon*, gene regulatory networks, nitrogen availability, root system architecture, transcriptomic plasticity

## Abstract

Nitrogen (N) form and availability shape the root phenotype by altering the root transcriptome and modulating root system architecture. However, it remains unclear whether these N-responsive architectural traits and their underlying regulatory networks are conserved or divergent between closely related grass species. Here, we compared root architectural and transcriptomic responses to nitrate and ammonium supply in *Brachypodium distachyon* and eight barley (*Hordeum vulgare*) genotypes under N starvation, optimal N supply, and high-N conditions. High-resolution root phenotyping revealed contrasting species-specific responses to N form. Barley generally showed enhanced lateral root elongation under nitrate but reduced total root length and limited lateral root proliferation under ammonium, indicating ammonium sensitivity. In contrast, *B. distachyon* maintained greater root architectural plasticity under ammonium, producing a highly branched and thinner root system. Cross-species module preservation and gene regulatory network analyses identified conserved hub genes, including FERONIA, peroxidase-like genes, CuAO-like genes, aspartyl proteases, and PEPC1-associated modules, as potential integrators of N signaling and root architectural remodeling in barley and *B. distachyon*. Taken together, our findings indicate that the two *Pooideae* species share conserved regulatory components but deploy them within distinct network contexts, leading to divergent root strategies in response to nitrate and ammonium. Ultimately, these results provide candidate genes and regulatory modules for improving root plasticity and N use efficiency in cereals.

## Highlight

- Cross-species root transcriptomics reveals conserved regulatory hubs and species-specific nitrogen responses controlling root architecture in barley and Brachypodium and Identified candidate genes for nitrogen-responsive root development across monocots.

## Introduction

The complexity of nitrogen (N) nutrition management in agriculture arises from the diversity of N forms in the soil, their chemical and physical properties and their continuous transformation through biological and physicochemical processes (Griffiths and York, 2020; Näsholm et al., 2009). In addition, plant-available N is inherently limited in the soil due to losses via leaching and denitrification, as well as adsorption to soil particles that can reduce its immediate accessibility for root uptake (Liu et al., 2025). In response to these highly dynamic but often N-limited environments, plants have evolved a wide range of physiological and morphological adaptations to efficiently acquire and utilize different N forms, which further contributes to the overall complexity of N nutrition management (Näsholm et al., 2009). Our current understanding of N dynamics highlights the critical role of root systems for efficient N uptake (Lynch, 2013). This makes root development and function essential research areas for enhancing nutrient acquisition and therewith plant productivity (Giehl and von Wirén, 2014). Furthermore, root phenological traits, particularly the level of investment in root biomass and architecture strongly influence N acquisition capacity (Hodge, 2004), but their impacts on the uptake of specific N forms and their genetic control are not well understood (Comas et al., 2013; Pereira et al., 2021).

*Brachypodium distachyon* is an established model for investigating root responses in monocot species. Previous studies showed that N availability affects root biomass, lateral root development and N-dependent root architecture in *Brachypodium distachyon* and other grasses (Jian-Bo et al., 2010; Poiré et al., 2014; Rouina et al., 2025). In the *B. distachyon* inbred line Bd21-3, contrasting architectural and metabolic responses upon application of different N forms were associated with candidate regulators, including aspartic proteases and phosphoenolpyruvate carboxylase 1 (PEPC1) (Rouina et al., 2025). Given the fundamental architectural differences between monocot and dicot root systems, direct comparisons among closely related monocot species are needed to determine whether N-responsive gene–trait relationships are conserved (Huang et al., 2025; Smith and De Smet, 2012).

Growing evidence from within-monocot comparisons (maize vs. sorghum; Du et al., 2020) supports the existence of conserved transcriptional modules controlling nitrate responses. However, to the best of our knowledge, a direct comparative analysis of N-responsive regulatory networks within the *Pooideae* and especially between barley and *B. distachyon* has not yet been performed. Comparative genomic analyses have demonstrated that *B. distachyon* exhibits a greater number of conserved syntenic loci with barley (14,422) than other grasses such as rice (12,093) and sorghum (11,887), underscoring its suitability as a model for *Triticeae* subfamily (Mayer et al., 2011). This close phylogenetic relationship between barley (*Hordeum vulgare*) and *B. distachyon* within the *Pooideae* subfamily offers therefore an especially powerful system for dissecting conserved regulatory networks relevant to other *Triticeae* crops as well (Mayer et al., 2011).

Despite the phylogenetic proximity between barley (*Hordeum vulgare*) and *B. distachyon*, they exhibit distinct differences in root phenotype (Watt et al., 2009). At germination, barley typically produces up to six seminal roots emerging from the embryo, establishing a robust and fibrous early root system (Hochholdinger et al., 2004; Singh et al., 2010; Watt et al., 2008, Watt et al., 2009). In contrast, *B. distachyon* initiates only a single primary root from the base of the embryo, similar to rice and maize (Vogel et al., 2009; Watt et al., 2008). Furthermore, *B. distachyon* generally lacks scutellar node-derived axile roots under standard conditions (Kirschner et al., 2017; Watt et al., 2009). The difference between barley and *B. distachyon* continues in the pattern of nodal root development. In barley, nodal roots emerge relatively early and vigorously from multiple nodes, supporting rapid soil exploration. In *B. distachyon*, however, nodal root emergence is delayed, typically initiated at the coleoptile node around the third leaf stage, with a more gradual development compared to barley (Watt et al., 2009). Despite these early differences, both species develop mature root systems that are dominated by lateral roots, with branched roots accounting for more than 90% of total root length (Watt et al., 2009). While these architectural differences are well described at the morphological level, it remains largely unknown whether the underlying transcriptional regulatory networks controlling N-responsive root development are conserved or are species-specific between barley and *B. distachyon*. In particular, a direct comparative transcriptome and network-level analysis of N-dependent root plasticity between these two closely related species has not yet been performed, to the best of our knowledge.

In this study, we compared root architectural and transcriptional responses to ammonium and nitrate availability in the *B. distachyon* inbred line Bd21–3 and eight barley inbreds. We hypothesized that the two species share a conserved core of N-responsive transcriptional mechanisms but differ in how these mechanisms regulate root system architecture under ammonium and nitrate nutrition. We therefore aimed to characterize species- and genotype-dependent root responses, identify conserved and divergent transcriptional modules, and prioritize candidate regulators associated with N-dependent root architectural plasticity.

## Material and methods

### Plants growth conditions

Our study was based on the inbred line *B. distachyon* Bd21-3. In addition, eight barley (*Hordeum vulgare*) genotypes were selected from the parental inbreds (Weisweiler et al., 2019) of the double round robin population (Casale et al., 2022) based on results from a preliminary screening experiment for variation in their RSA (long, medium, and short root systems) and their range of architectural responses to standard ammonium and nitrate treatments.

Seed sterilization procedures were optimized for barley and Bd21-3. The seeds were surface-sterilized for 1 minutes using 6% sodium hypochlorite (NaOCl) with 0.1% Triton X-100 under sterile conditions, followed by five washes with autoclaved distilled water. The Bd21–3 seeds were germinated directly on agar plates containing one-third strength Hoagland medium (Hoagland, 1920). Sterilized barley seeds were placed on moist germination paper rather than agar medium to better support their larger seed size and their root growth. Barley and Bd21–3 seeds were stratified at 4 °C for 3 days in darkness at 18 °C/16 °C (day/night). After germination, one barley and two Bd21–3 seedlings per plate were transferred to separate 250 ml customized Petri dishes containing one-third strength Hoagland medium supplemented with either ammonium or nitrate as the sole N source. The barley and *B. distachyon* seedlings were cultivated for 10 and 16 days, respectively, using the GrowScreen-Agar phenotyping system under a 14 h light/10 h dark photoperiod. A fully randomized block design was used for the experiment and three concentrations (0, 1.5, and 6 mM) (**Supplementary Figure 1**). The N levels were selected to represent biologically distinct N regimes, including starvation (0 mM), optimal (1.5 mM, approximate optimal supply based on the estimated plant N requirement), and high-N conditions (6 mM). Nevertheless, differences in seed reserves during germination and early seedling establishment may partly buffer or delay the response to external N limitation. To minimize this potential bias, plants were grown for a longer period (10 for barley and 16 days for *B. distachyon*). At these stages, the initial seed reserves were expected to be largely depleted, and plant growth was therefore assumed to depend primarily on external N supply.

For our phenotypic characterization, each Petri dish was considered as one independent experimental unit. Because *B. distachyon* seedlings are substantially smaller and produce less biomass than barley seedlings, two Bd21–3 plants were grown per dish, whereas one barley plant was grown per dish. The two Bd21–3 plants within a dish were treated as subsamples rather than independent biological replicates, and their phenotypic measurements were averaged to obtain one value per experimental unit. Each treatment included four independent dishes per experimental repetition, and the experiment was conducted four times. Thus, both species were represented by 16 independent experimental units per treatment.

A separate experiment was performed with Bd21–3 plants for RNA extraction. For each RNA-seq biological replicate, roots from nine plants were pooled to obtain sufficient tissue. In contrast, the barley plants used for phenotyping were also used for tissue collection, as one barley plant provided sufficient root material for RNA extraction. The RNA-seq replication and sample preparation are described separately below.

The above mentioned standard 1/3 Hoagland medium includes KNO_3_, Ca (NO_3_) 2(H_2_O), MgSO_4_, KH_2_PO_4_, trace elements (MnCl_2_ 4 H_2_O, CuSO_4_ 5 H_2_O, ZnSO_4_ 7 H_2_O, H_3_BO_3_, Na_2_MoO_4_ 2 H_2_O), and [Fe_3_^+^-EDTA]. To apply ammonium and nitrate as solo N sources in the medium at different concentrations, we modified the standard solution by removing Ca (NO_3_) 2(H_2_O) for nitrate application and replacing KNO_3_ with (NH_4_) 2SO_4_ for ammonium application. To compensate for the absence of potassium and calcium from the modified medium, we added K_2_SO_4_ and CaCl_2_ to the 1/3 Hoagland medium in the corresponding concentrations.

### Root phenotyping and sampling

RSA was assessed using the GrowScreen-Agar phenotyping platform, which uses three cameras to capture images from the front, top, and root perspectives. The root was collected based on the protocol described before (Rouina et al., 2025). Roots from all plants in each replicate were harvested and immediately frozen in liquid N. Imaging was conducted every two days throughout the experimental period. Image files were manually processed using Adobe Photoshop to crop and enhance contrast before quantitative analysis. Root traits were extracted using RhizoVision Explorer software (Seethepalli et al., 2021), providing standardized phenotypic data across both species.

### Statistical analyses of phenotypic observations

Mean trait values were computed for each treatment, and the variation among the mean trait values of the two N treatments and concentrations was evaluated using analysis of variance (ANOVA). When the ANOVA indicated significance at the 5% probability level, the Tukey’s HSDtest was used to assess differences between treatment and concentration means. The effects of N concentration, N form, genotype, and their interactions on root traits were evaluated using a three-way ANOVA performed separately for the barley dataset. For Bd21-3, the average measurement of the two plants grown within each dish was used as a single observation in the statistical analysis,as was explained above. All analyses were performed in R.

### RNA extraction and transcriptome analysis

The frozen root tissue was ground in liquid N into a fine powder using a homogenizer. Next, 450 µL of RTL buffer containing 10 µL of beta-mercaptoethanol was added to the tube and stirred for 5 minutes. The samples were then transferred to a RNeasy Mini spin column placed in a 2 mL collection tube and centrifuged at 10,000 x g for 10 seconds. The flow-through was transferred to a new RNase-free tube, and 202 µL of absolute ethanol was added. The extracted RNA was used for preparing a sequencing library using the Ion Total RNA-Seq Kit v2 following the protocol from Life Technologies, USA. Libraries were paired-end sequenced using Illumina short reads with 150 bp. Quality assessment of the sequencing data was performed using FAST-QC (Andersson et al., 2010). After adapter trimming and quality filtering, an average of 22–26 million clean reads per library were retained. The retained reads were then mapped to the corresponding reference genome of *B. distachyon* (Brachypodium_distachyon_v3.0_genomic) and *Hordeum vulgare* (Genome assembly MorexV3_pseudomolecules_assembly) using HISAT2 software (Kim et al., 2015). Subsequently, HTseq software was used to quantify the number of aligned reads that overlap with the exons of each gene.

To correct for potential batch effects in the barley RNA-Seq data, we applied the “ComBat_seq” function from the “sva” R package prior to downstream analyses (Zhang et al., 2020). In order to do so, batch information was provided as a categorical variable. We fitted a full model, which helps to protect against over-correction in the absence of batch effects. Shrinkage was disabled for both the dispersion and the empirical Bayes adjustment to retain sensitivity to subtle transcriptomic differences (Nygaard et al., 2016; Zhang et al., 2020). This normalization procedure was applied independently to the read count datasets from *B. distachyon* and barley.

The “edgeR” package was then utilized to identify differentially expressed genes (DEG) based on a significance threshold of P value < 0.05 and a false discovery rate threshold of FDR < 0.1. Using a general linear model, we compared low versus moderate, low versus high, and moderate versus high levels of nitrate and ammonium and within the genotype level of barley. Gene ontology (GO) enrichment for biological processes was performed for DEGs using the “enricher” function of the “Biomart” library (Durinck et al., 2009) and visualized using clusterProfiler package in R (Yu et al., 2012). No independent qPCR validation of the RNA-seq expression patterns or functional validation of the predicted TF–target relationships was performed in this study.

### Weighted gene co-expression network analysis

The normalized RNA-seq data were analyzed using the weighted gene co-expression network analysis (WGCNA) package in R to explore the correlation between transcriptomes across various treatments (Langfelder and Horvath, 2008). Lowly expressed genes were filtered out using the “filterByExpr” function from the “edgeR” package, which retains genes with counts above a dynamic CPM threshold in a minimum number of samples that are sufficient for statistical analyses (Chen et al., 2016). Filtered genes were employed to construct weighted gene co-expression networks. Pearson correlation matrices for gene expression were computed for each network and transformed into connection strength matrices using an appropriate power function to ensure scale-free behavior. These connection strengths were then converted into a Topological Overlap Measure (TOM).

TOM, combined with average linkage hierarchical clustering, was applied via the DynamicTree Cut algorithm to identify distinct co-expressed gene modules. To link these modules to phenotypic observations, expression data were integrated with phenotypic responses across all treatments. Modules with an absolute correlation coefficient ≥ 0.4 and a p-value ≤0.05 were considered and selected for further analyses. Within each significant module, module membership and gene significance were calculated for all genes. Hub genes were defined as those with high intramodular connectivity, specifically genes showing strong correlation with the module eigengene (high module membership) and high correlation with the trait of interest (high gene significance). These hub genes were selected for further analysis and GRN reconstruction. Species-specific co-expression networks were constructed for each of the two species. The *B. distachyon* network comprised 21 RNA-seq samples from the single inbred line Bd21-3, whereas the barley network comprised 168 samples from eight genetically distinct barley genotypes, with 20 samples contributed by each genotype. The larger barley sample number resulted from the inclusion of multiple genotypes to capture intraspecific variation. The barley and *B. distachyon* expression datasets were not pooled during network construction.

### Cross-species ortholog mapping and expression integration

Orthologous gene mapping between *Hordeum vulgare* and *B. distachyon* was established using a multi-step integration workflow implemented in R. Functional orthology relationships between species were retrieved from the g:Profiler ortholog dataset, which provides curated gene-to-gene homology assignments across genomes. Only columns corresponding to source gene identifiers and barley ortholog assignments were retained, and missing annotations were converted to missing values.

The ortholog reference table was merged with the expression dataset by gene identifier to preserve all expressed Bd21–3 genes with available barley orthologs. Genes lacking ortholog annotations were excluded from further analyses. Because multiple Bd21–3 genes may map to the same barley ortholog (many-to-one relationships), duplicate ortholog groups were resolved by ranking candidate transcripts according to their total expression across all samples. For each barley ortholog group, the Bd21–3 gene with the highest cumulative expression was retained as the representative ortholog, thereby minimizing the impact of low-confidence or weakly expressed paralogs.

To enable cross-species network comparison and module projection, barley gene identifiers were harmonized using a customized NCBI-to-Ensembl mapping table, ensuring that each remaining gene corresponded to a single standardized barley locus. The final curated dataset represents a set of one-to-one and best representative many-to-one orthologous gene pairs linking Bd21–3 transcripts to their corresponding barley loci, providing a harmonized expression matrix suitable for co-expression analysis and GRN inference across species.

### Module preservation analysis

The independently constructed species-specific networks were subsequently compared through orthology-based module-preservation analysis. Differences in sample size and represented genetic diversity between the two networks were considered when interpreting the preservation statistics. To assess the conservation of co-expression network modules between Bd21–3 and barley, we conducted a module preservation analysis using the WGCNA package in R. Expression matrices were transposed, and their quality was checked to remove lowly expressed genes as well as retain only high-quality genes and samples using approaches similar to those explained in previous sections. Bd21–3 was used as the reference in the analysis, and preservation of modules was tested in barley using “modulePreservation” function in the “WGCNA” package. The analysis used 1000 permutations to compute module preservation statistics. The goal was to identify co-expressed gene modules with conserved regulatory roles and to uncover key genes and transcription factors (TFs) involved in controlling RSA under varying N concentrations and forms. Module preservation was evaluated using both Zsummary and medianRank statistics from WGCNA. On the other hand, we used the expression of hub genes and GO term enrichment analysis to characterize the conserved and unique expression responses in barley inbreds and Bd21-3.

### Gene regulatory network analysis

Candidate target genes were defined in our study as hub genes from preserved co-expression modules that were significantly associated with total root length (TRL), lateral root length (LRL), or root diameter. Candidate transcription factors (TFs) and their corresponding DNA-binding motifs were obtained from the CIS-BP species-specific motif collections for *B. distachyon* and barley. The 1,500-bp genomic region upstream of each candidate target gene was defined as its promoter and scanned for TF-binding motifs using RSAT.

To construct expression-supported gene regulatory networks, a candidate TF–target relationship was retained only when it was supported by both promoter motif evidence and expression correlation. Pearson correlation coefficients (PCCs) were calculated independently for each species between the expression profiles of candidate TFs and their putative target genes. Edges were retained.

## Results

### Root phenotypic and transcriptomic variation across barley genotypes and Bd21-3

#### Root phenotype

To investigate root system responses to different N forms and concentrations, we analyzed TRL, LRL, RD, and DW across eight barley inbreds and the *B. distachyon* inbred line Bd21-3. In barley, TRL was significantly affected by N concentration (p = 2.02 × 10^-9^), N form (p = 0.0002), and genotype (p = 2.47 × 10^-^¹²). Furthermore, a significant interaction between N concentration and genotype (p = 0.0027) was observed, suggesting that genotypic response to N level is not uniform across genotypes. The interaction between N concentration and source was also significant (p = 0.000229), highlighting a complex response pattern based on both N form and concentration. In Bd21-3, both N concentration (p = 2.72 × 10^-5^) and form (p = 0.0006) significantly influenced TRL, however no significant interaction was observed between these two factors. Across both species, increasing N concentrations led to a general decline in TRL, highlighting a potential suppressive effect of high N availability on TRL (**Figure 1A**).

**Figure 1:**
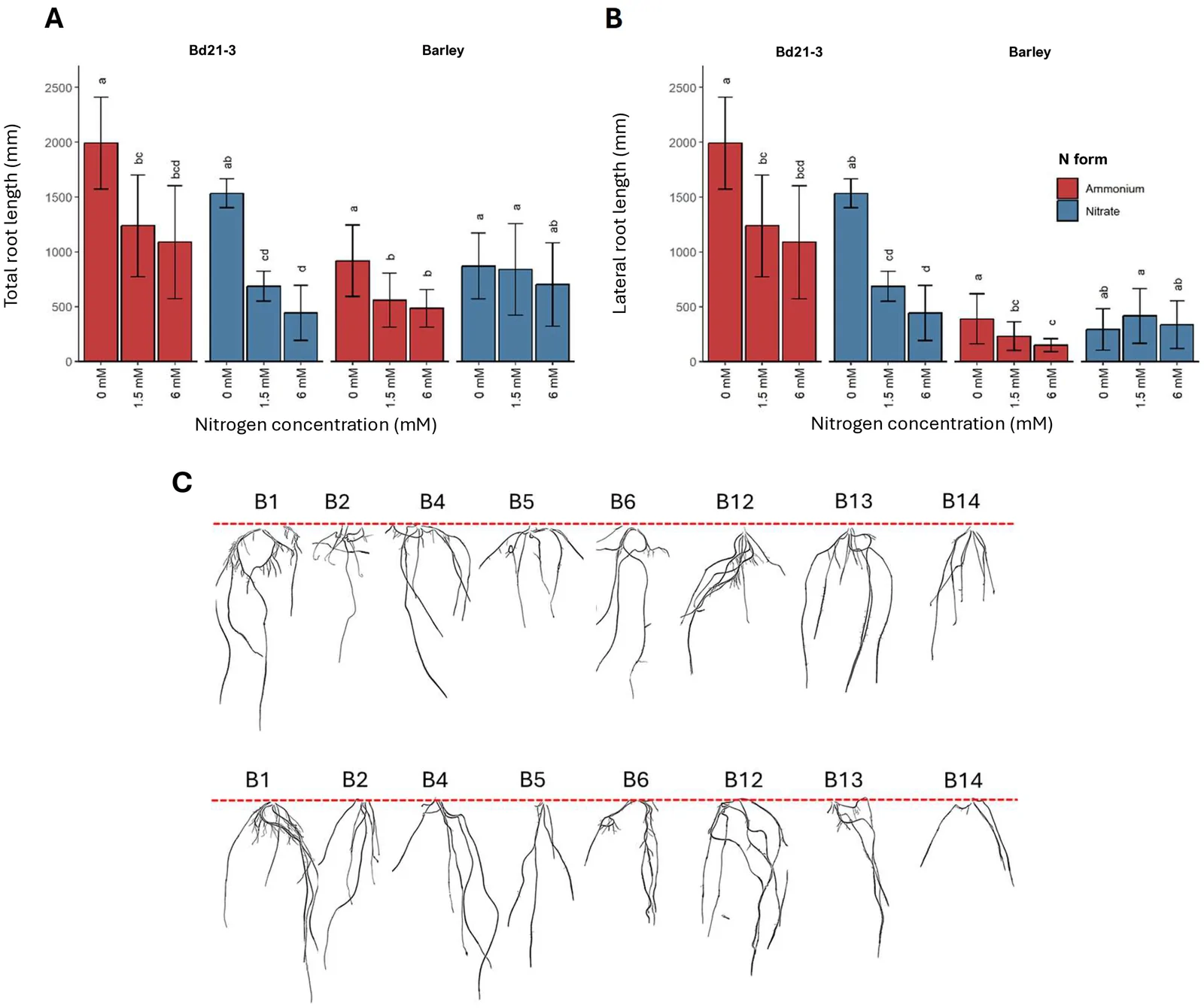
Root-length responses of *Brachypodium distachyon* and barley to nitrogen source and concentration. **(A)** Total root length (TRL) and **(B)** lateral root length (LRL) of Bd21-3 and barley (mean across eight genotypes) under ammonium and nitrate supply at 0, 1.5, and 6 mM. For Bd21-3, factorial ANOVA revealed significant main effects of N concentration and N source on both TRL and LRL, but no significant concentration x N- source interaction. **(C)** Representative root systems of barley genotypes grown under 1.5 mM ammonium (upper panel) or nitrate (lower panel). Error bars represent standard deviations. Different letters indicate significant differences among treatments according to Tukey's HSD test (P < 0.05). Complete ANOVA results are provided in **Supplementary Table 1**.

In addition, we observed on average across all barley genotypes an increased LRL in response to nitrate treatments compared to ammonium, with peak values occurring at 1.5 mM nitrate followed by a moderate decline at 6 mM (**Figure 1B**). In contrast, Bd21–3 exhibited the opposite trend, with greater LRL with ammonium treatments compared to nitrate treatments. Biomass measurements indicated that despite higher TRL in Bd21-3, barley produced higher overall DW (**Supplementary Figure 2**). Despite both species showed higher TRL and LRL under N-free conditions compared to N containing treatments, barley developed markedly thicker roots, with diameters being approximately three times greater than those of Bd21-3 (**Supplementary Figure 2**).

Barley showed substantial intraspecific variation in response to N treatments (**Figure 1C**). Notably, TRL varied significantly among genotypes across concentrations (p = 0.002682) (**Supplementary Figure 2**). However, no statistically significant differences were observed for LRL for the same comparisons (**Supplementary Figure 2**), indicating that LRL is less dependent on genotypic variation compared to TRL. Interestingly, most barley genotypes displayed comparable root and shoot DW between ammonium and nitrate treatments which illustrates a certain level of plastic growth regardless of N form (**Supplementary Figure 2**).

#### Differential gene expression analysis

In order to compare barley’s and Bd21-3*’s* gene expression in response to varying N forms and concentrations, we performed a comparative analysis of DEGs. Hierarchical clustering of transcriptomic data showed a dominant genotype-specific effect in barley (**Figure 2**). This suggests that despite external N fluctuations, the genotype identity plays a principal role in shaping gene expression. However, for *B. distachyon* where only one genotype was examined, we observed a clear cluster of N concentrations and N forms (**Supplementary Figure 3**). We observed for this species a stronger transcriptional response to ammonium and a greater number of DEGs under ammonium treatments compared to nitrate, in particular, low vs high or moderate ammonium (**Supplementary Figure 3**). Similarly, when considering barley at the species level (*i.e.* on average across all eight genotypes), change in ammonium supply induced a stronger transcriptional response than changes in nitrate. Nevertheless, this response was also strongly modulated by genotype. Several barley genotypes (B1, B4, B6, B12, and B13) exhibited relatively stable DEG counts across low to moderate and moderate to high nitrate concentrations, as well as across ammonium treatments (**Supplementary Figure 3**). In contrast, genotype B14 exhibited minimal transcriptional response to N application, potentially indicating a reduced sensitivity or stable N status under varying conditions. B5 and B2 demonstrated a pronounced increase of gene expression from moderate to high ammonium concentrations, indicating an ammonium-induced transcriptional reprogramming in these genotypes.

**Figure 2:**
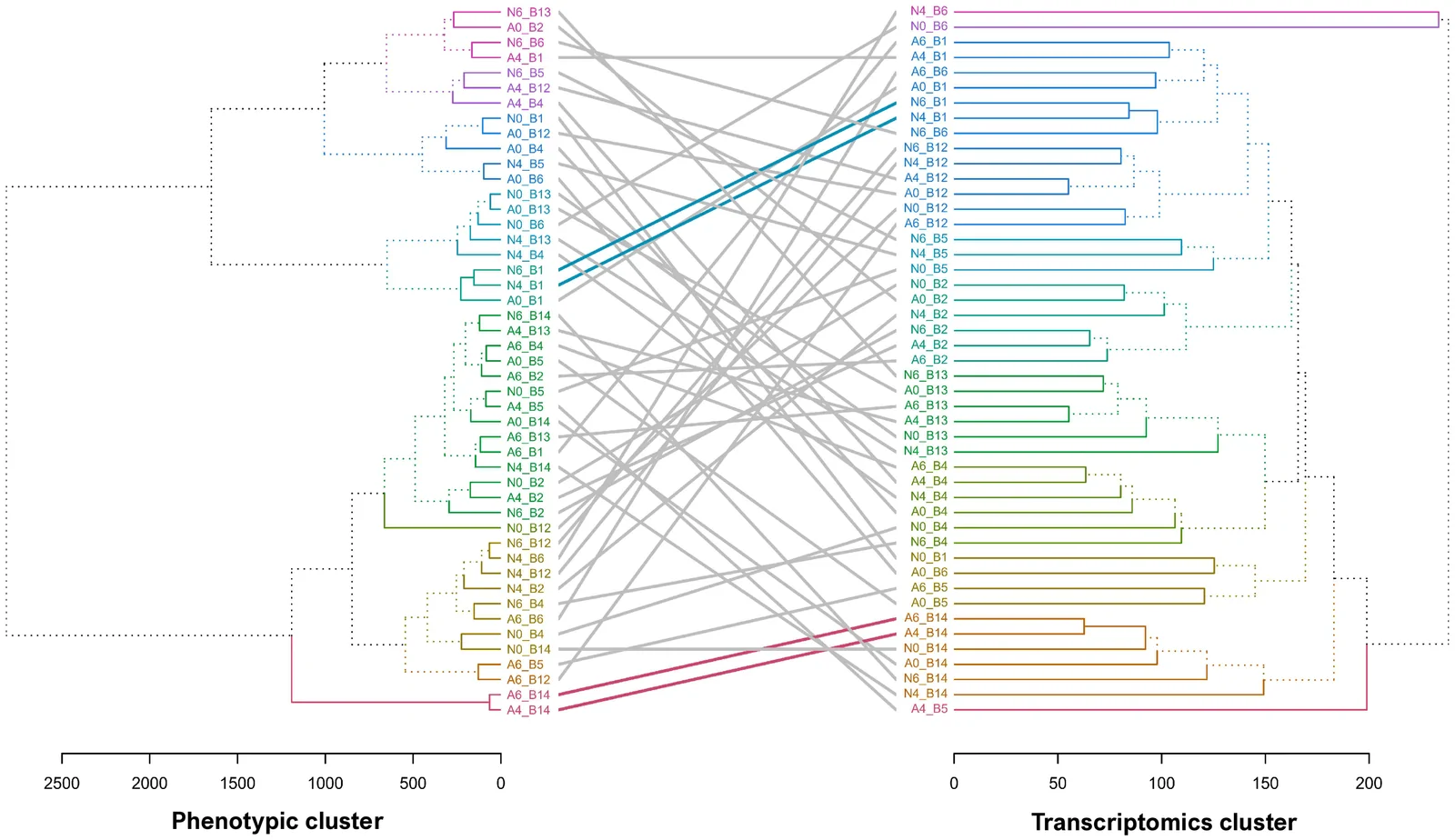
Dendrogram of hierarchical clustering of root phenotype (left) and transcriptome (right) profiles for barley genotypes under different N treatments. Each label represents the N treatment (A, ammonium; N, nitrate; followed by concentration in 0 = 0 mM, 4 = 1.5 mM and 6 = 6 mM) and genotype. Samples are color-coded by major cluster groups, illustrating similarities between phenotypic and root transcriptome profiles.

#### Weighted gene co-expression network analysis

To identify the gene co-expression clusters, we performed WGCNA separately for barley and *B. distachyon* and identified 35 and 32 gene co-expression modules, respectively, which were assigned different colors to identify them unequivocally. Among these, the Hv-purple, Hv-blue, Hv-cyan, and Hv-red modules showed high correlations with traits including TRL, LRL, mean lateral root length, and average RD in barley (**Supplementary Figure 4**). In Bd21-3, Bd-light-green, Bd-dark-red, Bd-yellow, and Bd-brown showed high correlations with TRL, LRL, root hair length and density, and plant N content in *B. distachyon*. The DEG analysis further revealed that the expression of the hub genes of these modules in barley showed similar expression patterns in response to N application compared to that of Bd21-3 (**Figure 3**).

**Figure 3:**
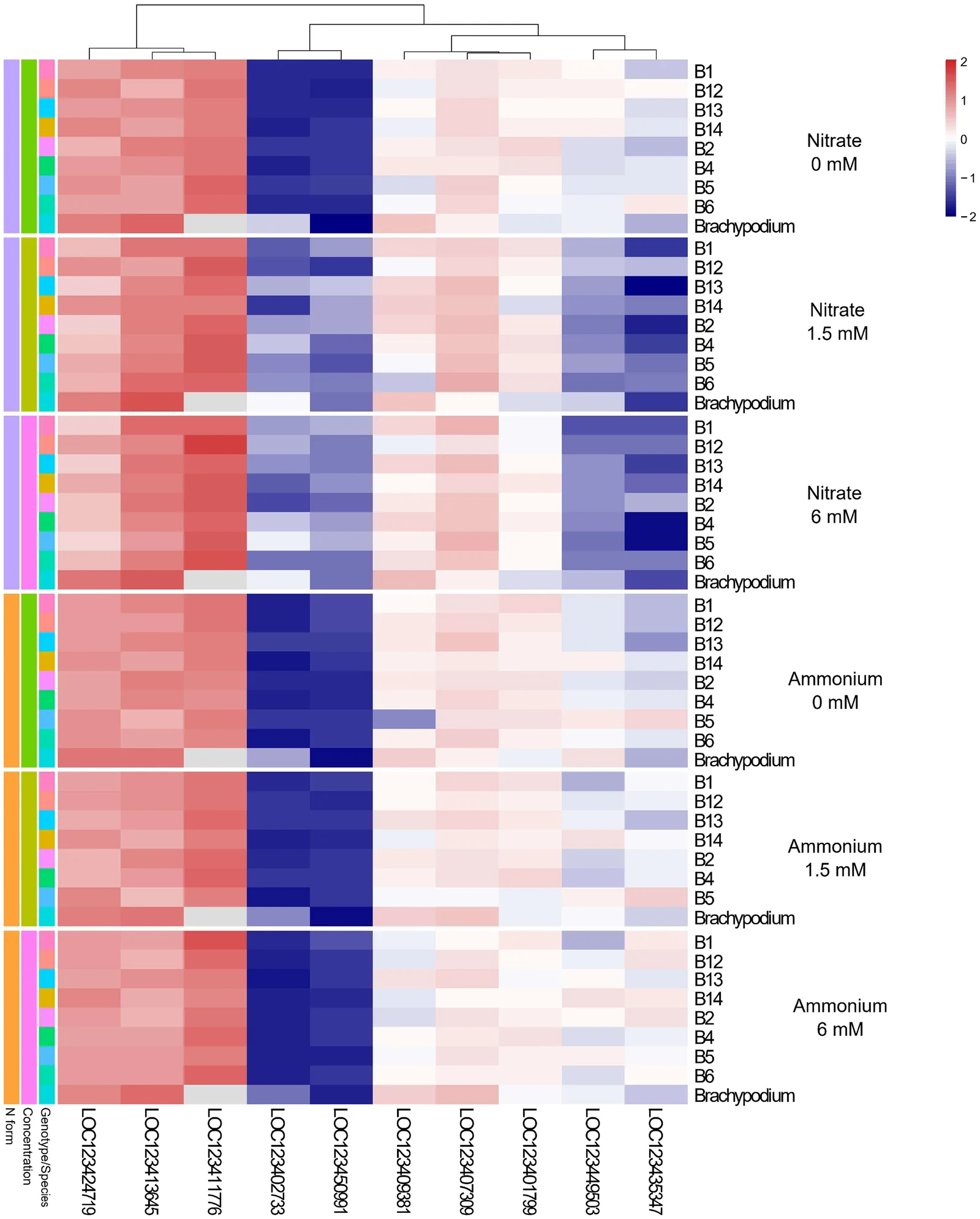
Expression patterns of barley hub genes, which are associated with root traits across nitrate and ammonium treatments at three concentrations (0, 1.5, and 6 mM). Rows represent barley genotypes and *Brachypodium distachyon (Bd21-3)*, and columns represent hub genes. Hierarchical clustering was applied to genes based on expression similarity. The color scale indicates normalized expression values, with blue representing low expression and red representing high expression.

The integration of DEG and WGCNA revealed that a substantial fraction of the significantly expressed genes among different N concentrations was part of these co-expression modules which highly correlated with TRL and LRL (Hv-Cyan and Hv-Red). This pattern was observed across both species and suggests a conserved network-level regulation of root architectural traits under contrasting N availability. GO enrichment analysis of these DEG-containing modules revealed a clear functional stratification to regulatory and metabolic processes across species. These genes that correlated with TRL and LRL were significantly enriched for nitrate transport, stress response, and RNA modification pathways, indicating root architecture alteration in response to N is governed by structured regulatory networks rather than as isolated signals (**Figure 4**). Upregulated genes in Hv-Cyan and Hv-Red modules in low ammonium were mainly associated with stress-response pathways versus high ammonium application. In contrast, downregulated genes were enriched for primary metabolism such as carbon fixation, amino acid metabolic process, and general metabolic processes, suggesting a reallocation of cellular resources under N stress or limitation (**Figure 4**). While the core regulatory themes were shared between barley and Bd21-3, subtle differences in enrichment strength and module composition suggest a species-specific tuning of N-responsive gene networks.

**Figure 4:**
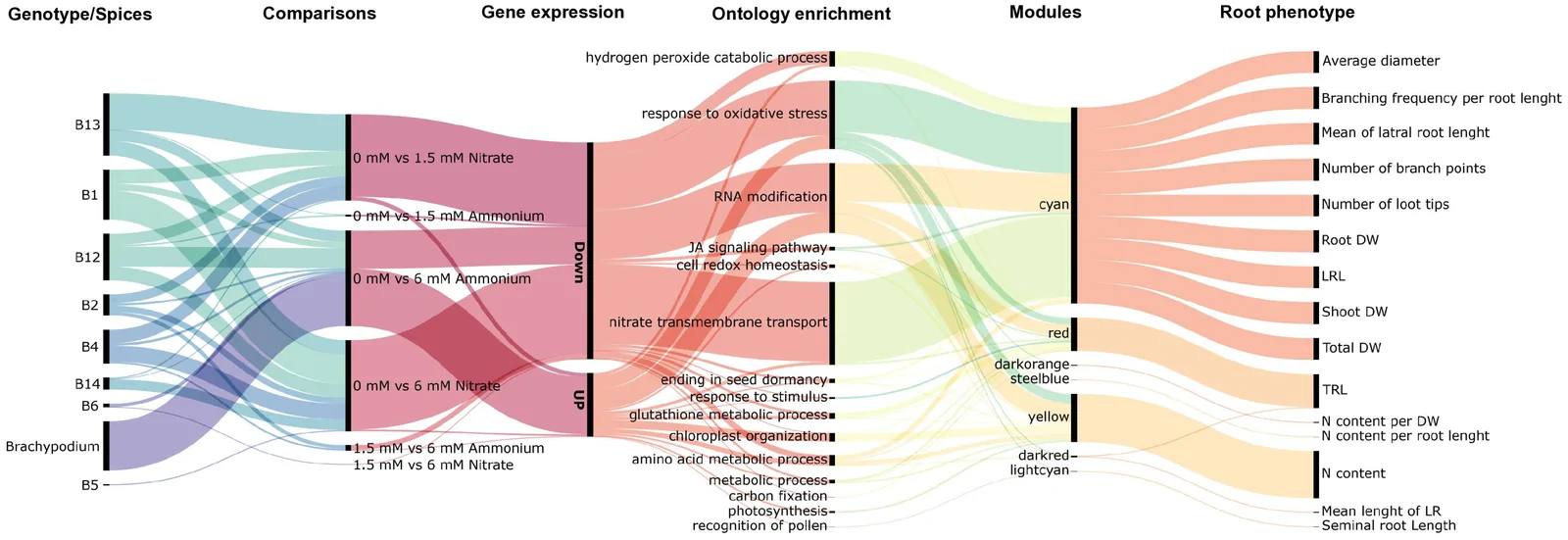
Integrated Sankey diagram illustrating the relationships between genotype/species, nitrogen-related comparisons, differential gene expression, gene ontology (GO) enrichment, WGCNA modules, and associated root phenotypes. Here, weighted gene co-expression network analysis (WGCNA) modules in both species were filtered based on significantly expressed genes across different comparisons and GO enrichment results were integrated with correlations to root phenotypes into a single visualization. Flows connect barley genotypes and *Brachypodium* to specific nitrate and ammonium contrasts, followed by gene expression responses, enriched biological processes, corresponding co-expression modules, and their significantly correlated root traits. The width of each flow reflects the relative contribution or number of genes shared between categories.

### Module preservation and biological pathway conservation across barley and *B. distachyon*

#### Module preservation

To investigate the evolutionary conservation of N-responsive regulatory modules, we performed a module preservation analysis as well as a regulatory network comparison between barley and *B. distachyon*. Several modules, namely Hv-blue (including 4200 genes), Hv-saddle-brown (including 65 genes), and Hv-green-yellow (including 351 genes), Hv-tan (including 197 genes), Hv-salmon (including 189 genes), and Hv-purple (including 360 genes) from the barley network had both high Zsummary values and low medianRank scores, identifying them as the most preserved modules in Bd21–3 gene co-expression network (**Figure 5A**). In contrast, the Hv-dark-red, Hv-dark, Hv-olive-green, and Hv-dark-turquoise modules showed the lowest preservation in Bd21-3. The majority of genes from both preserved and non-preserved barley modules were distributed to the Bd-blue, Bd-green, Bd-turquoise, Bd-dark-gray, Bd-tan, and Bd-brown modules in Bd21-3 (**Figure 5B**).

**Figure 5:**
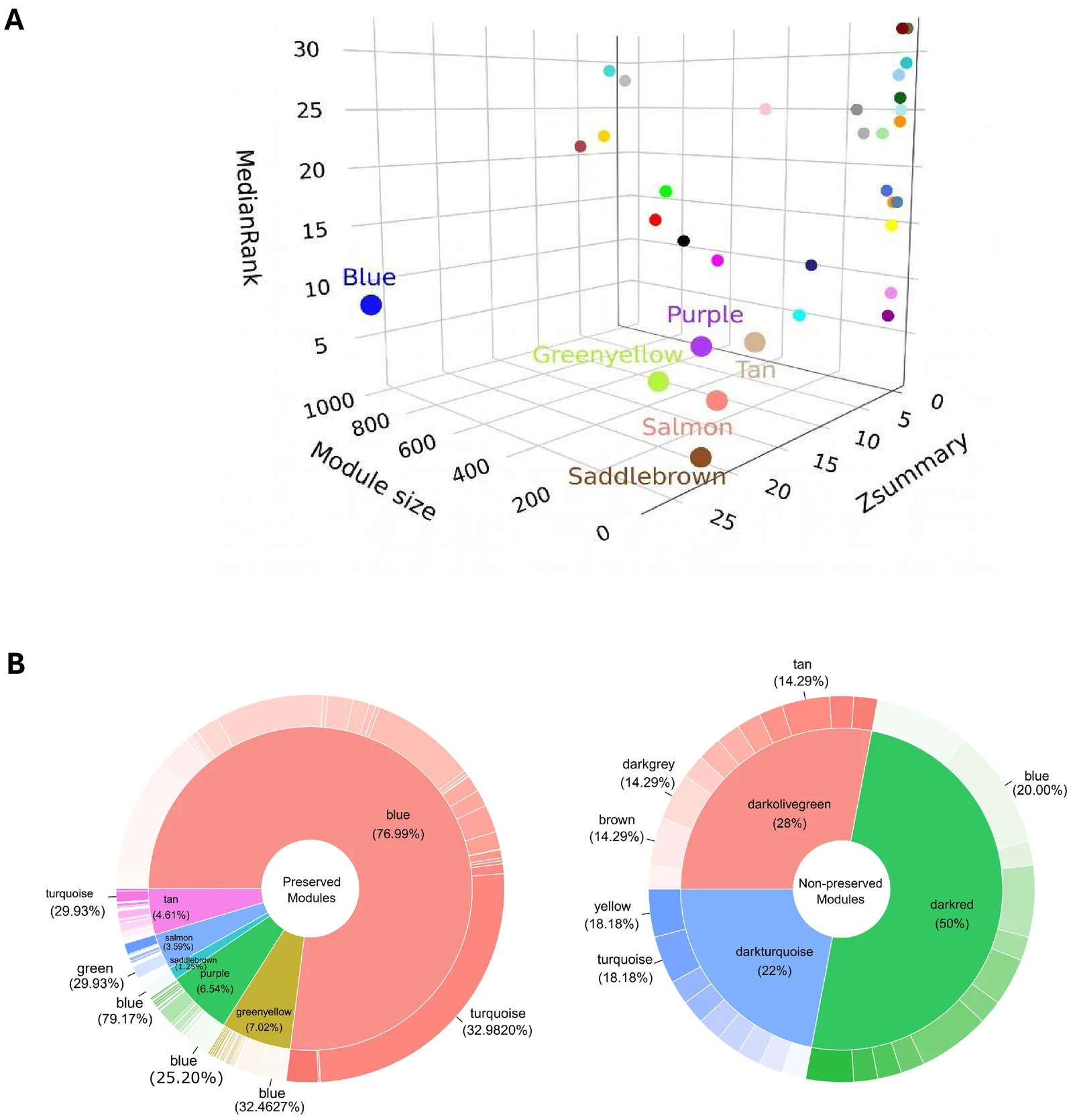
Module preservation statistics for the comparison of barley vs *Brachypodium distachyon* as identified from co-expression network analysis (WGCNA). **(A)** Three-dimensional scatter plot summarizing WGCNA module preservation statistics, with modules positioned based on module size, median rank, and Z-summary score. Higher Z-summary values indicate stronger module preservation across species, while lower median rank values reflect more reliable preservation independent of module size. Preserved modules (blue, turquoise, greenyellow, purple, tan, salmon, and saddlebrown) are highlighted and labeled. **(B)** The relative distribution of genes among preserved (left) and non-preserved (right) modules in Bd21–3 co-expression modules. The inner layer (inside) corresponds to barley modules, while the outer layer (outside) represents Bd21–3 modules.

#### Nitrate triggers a conserved metabolic response in both species

GO enrichment analysis revealed distinct functional themes of the modules that are preserved across barley and Bd21–3 in response to N source and concentration change (**Supplementary Data Sheet 1**). The Hv-tan module was mainly associated with intracellular transport, vesicle trafficking, and protein turnover (**Supplementary Data Sheet 1**). In contrast, the Hv-salmon and Hv-saddle-brown modules were enriched for trehalose metabolism which is linked to stress tolerance and energy storage as well as cytosolic localization and sugar response (**Supplementary Data Sheet 1**). The Hv-green-yellow module showed an enrichment for cell wall biosynthesis and anatomical development.

To better understand how these preserved modules transcriptionally respond to nitrate supply, we next examined the differential expression patterns of genes within these modules following nitrate application. When plants were exposed to nitrate (low vs moderate and low vs high concentrations), both barley and Bd21–3 showed an upregulation of genes involved in nitrate transport activity and the pentose phosphate pathway (PPP) (**Supplementary Data Sheet 1**). Although both nitrate and ammonium assimilation require energy, nitrate nutrition imposes an additional demand for reducing power. Ammonium can enter the GS/GOGAT assimilation pathway directly, whereas nitrate must first be reduced to nitrite by nitrate reductase and subsequently to ammonium by nitrite reductase. Together, these reduction steps require eight electrons before the resulting ammonium can be incorporated into amino acids. This additional reductant demand may explain the enrichment of the pentose phosphate pathway under nitrate nutrition in roots, where photosynthetic electron transport is unavailable. The oxidative pentose phosphate pathway provides NADPH in non-photosynthetic plastids. NADPH may support ferredoxin-dependent nitrite reduction through root-type ferredoxin–NADP(H) oxidoreductase and provide reducing power for other processes associated with nitrate assimilation. Ammonium nutrition bypasses nitrate and nitrite reduction and may therefore impose a lower demand for coordinated oxidative pentose phosphate pathway activity, although its assimilation through GS/GOGAT still requires ATP and reducing equivalents. However, because pathway activation was inferred from transcript enrichment rather than direct measurements of metabolic flux or NADPH production, this proposed mechanism requires further experimental validation. This observation indicates that genes embedded in conserved co-expression networks are transcriptionally activated in response to nitrate, linking network preservation with a conserved metabolic adjustment. In particular, the induction of nitrate transporters together with PPP-associated genes suggests a coordinated response that integrates nutrient acquisition with energy production and redox balancing.

Notably, only minimal DEG activity was observed between moderate and high nitrate concentrations in most barley genotypes, as well as in Bd21–3, indicating that the primary transcriptomic response is triggered at low thresholds of nitrate exposure.

#### Ammonium triggers species-specific and detoxification pathway

To determine how genes within the preserved co-expression modules respond to ammonium supply, we examined in analogy to what was described above for nitrate and their differential expression patterns under varying ammonium concentrations. For the ammonium treatment, comparisons between low vs moderate and low vs high ammonium concentrations revealed an upregulation of genes related to glutamine biosynthesis and amino acid transporter activity in both barley and Bd21-3 (**Supplementary Data Sheet 1**).

Thus, similar to the nitrate response, conserved modules contain genes that mediate a core N-assimilation program shared between the two species. However, species-specific differences became apparent at higher ammonium concentrations. While Bd21–3 did not exhibit pronounced additional transcriptional shifts between moderate and high ammonium levels, several barley genotypes showed further activation of stress-related genes and ion transporters, including transporters associated with cadmium and sulfate movement (**Supplementary Data Sheet 1**).

### Cross species preserved regulatory network

#### TF families

In addition to a WGCNA, a gene regulatory network analysis was performed to identify potential TFs binding to motif sites in the promoters of hub genes of the above-described modules. In addition, two motifs, M07266_3.00 and M06966_2.00, were among the most significantly enriched elements in the promoters of hub genes. These motifs were predicted using the RSAT matrix-scan tool (https://rsat.eead.csic.es/plants/matrix-scan_form.cgi) to bind transcription factors of the DOF family in barley and the LOB family in Bd21-3, respectively (**Figure 6**). These TF genes were themselves not expressed in our datasets. The above-mentioned TFs G2-like and C2H2 ranked highest in the networks of barley (betweenness centrality= 0.91 and degree= 7) and Bd21-3 (betweenness centrality= 0.31 and degree= 6) illustrating their likely role as regulatory hubs (**Supplementary Data Sheet 2**). Nevertheless, the networks of *B. distachyon* and barley differed in their topological structure. Barley exhibited a more evenly distributed connectivity pattern, with multiple TFs contributing to network cohesion. In contrast, Bd21–3 displayed a more centralized architecture characterized by fewer high-centrality regulators.

**Figure 6:**
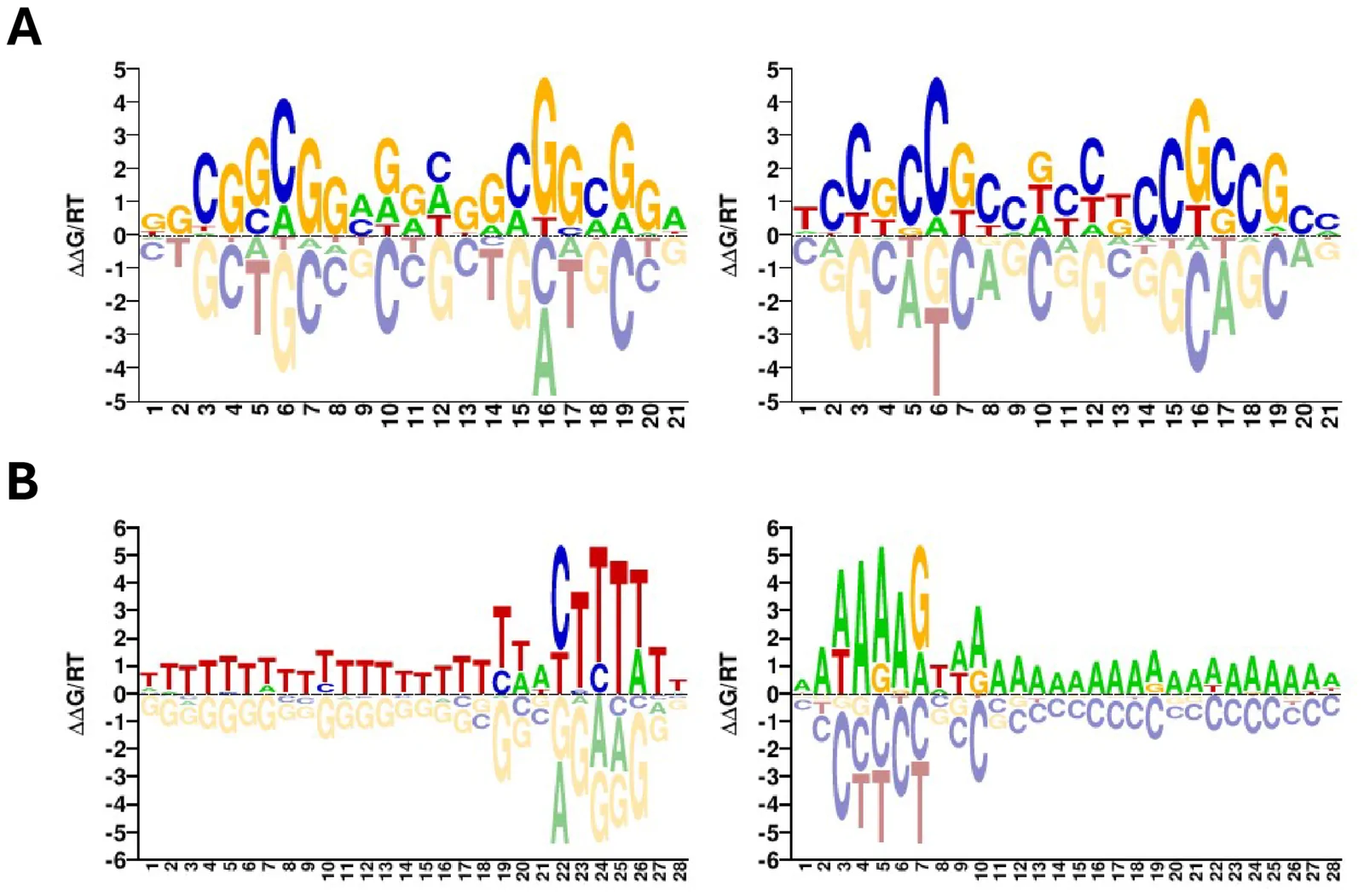
Enriched cis-regulatory motifs identified in the promoter regions of root phenotype–associated hub genes. **(A)** Sequence logos of the most significantly enriched motifs detected in barley hub gene promoters, including motif M07266_3.00, predicted to bind DOF transcription factors, forward (right), reverse (left). **(B)** Sequence logos of enriched motifs in *Brachypodium distachyon* (*Bd21-3*) hub gene promoters, including motif M06966_2.00, predicted to bind transcription factors of the LOB family forward (right), reverse (left). Motif enrichment was assessed relative to background promoter sequences, and sequence logos represent nucleotide frequencies at each position.

#### Target genes

Among the hub genes targeted by the previously presented TFs, several have confirmed functions in root development pathways and root phenotype regulation in species like *A. thaliana*, rice and maize (**Supplementary Data Sheet 3**). These include LOC123409381 (BRADI_3G41340v3, peroxidase 55-like) (Marzol et al., 2022; Mukherjee and Corpas, 2023), LOC123440783 (BRADI_1G63320v3, receptor-like protein kinase FERONIA) (Lin Liu et al., 2021), LOC123439773 (BRADI_2G09660v3, PRX72) (Du et al., 2020; Preiner et al., 2019; Prinsi and Espen, 2018), LOC123447059 (BRADI_1G24010v3, primary amine oxidase-like) (Qu et al., 2017), and LOC123447531 (BRADI_1G10630v3, actin-1) (Feng et al., 2022; Jedličková et al., 2022; Vaškebová et al., 2018).

Hub genes of the preserved module (Hv-purple) in the Bd21–3 network, namely LOC123409381, LOC123447059, LOC123440783, LOC123440079, LOC123407309, and LOC123447531 exhibited binding sites for the TFs from the bZIP, bHLH, MYB, NAC, and Trihelix families in their promoter regions (**Figure 7A**). In contrast, within the gene network of barley, only two genes, namely LOC123425423 from the Hv-saddle-brown module and LOC123413645 from the Hv-purple module, had predicted regulatory associations with HD-ZIP and NAC family transcription factors (**Figure 7B**). Although, homologs of LOC123425423 and LOC123413645 genes have known roles in *A. thaliana*, rice, and maize, their regulatory connections within N-responsive gene networks in barley and Bd21–3 are still uncharacterized. Genes in the Hv-purple module was enriched for anatomical structure development and cell wall biogenesis, highlighting their functional association with root structural remodeling (**Supplementary Data Sheet 4**).

**Figure 7:**
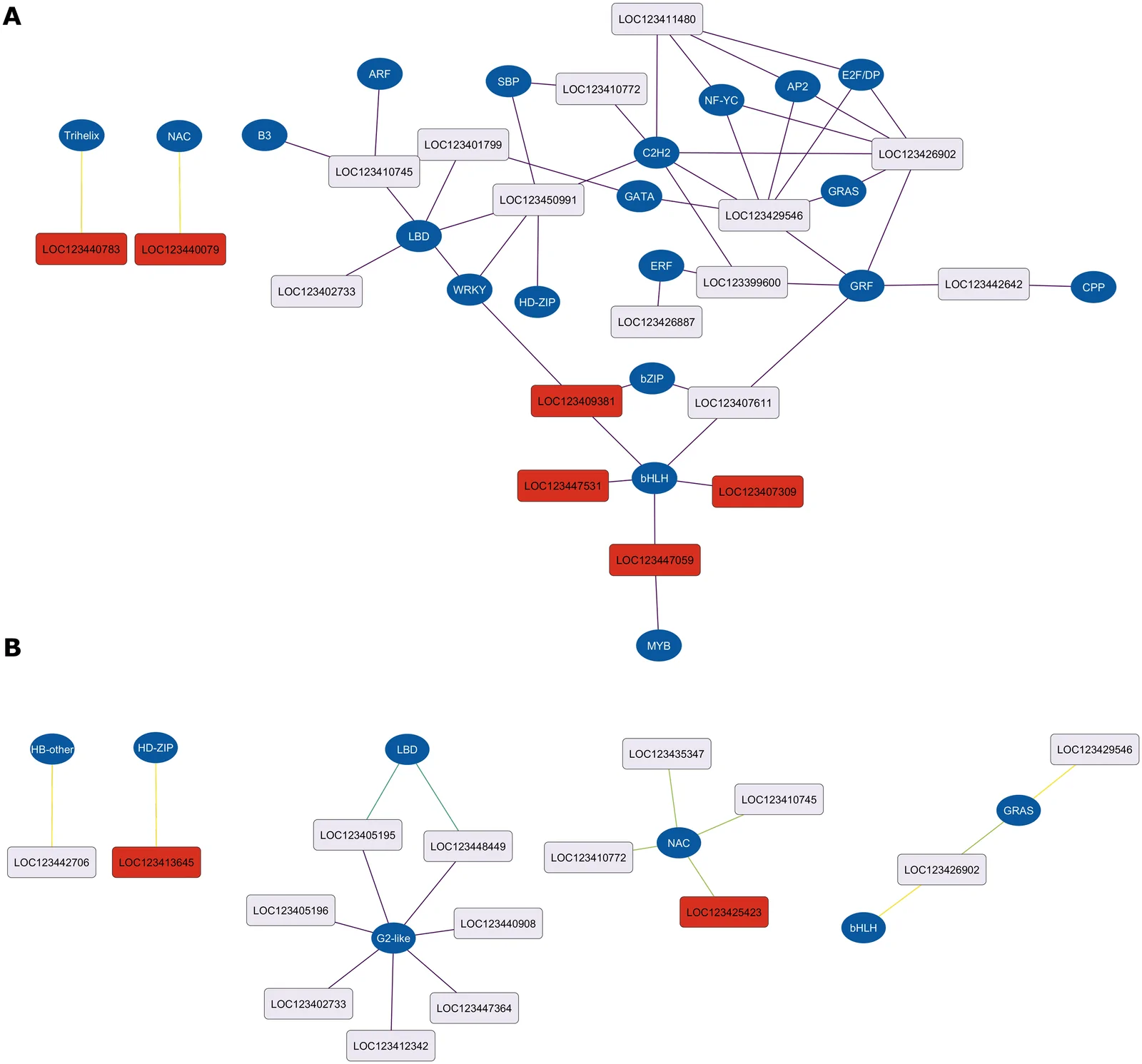
Root regulatory networks of hub genes of preserved modules among *Brachypodium distachyon* and barley. **(A)** Root gene regulatory network in *Brachypodium distachyon* (*Bd21-3*) constructed from hub genes of preserved modules identified in barley, together with three *Bd21–3* preserved hub genes. **(B)** Root gene regulatory network in barley constructed from hub genes of preserved modules, including the three conserved *Bd21–3* hub genes. Nodes represent genes or transcription factors (TFs), with TF families shown as blue circles and target genes shown as rectangular nodes. Red nodes indicate hub genes belonging to preserved co-expression modules across species. Edges represent predicted regulatory interactions between TFs and target hub genes based on motif enrichment and network inference. Network topology highlights conserved regulatory relationships underlying root phenotypic responses across two species.

The peroxidase 55-like gene (LOC123409381 highly correlated with mean lateral root length) was induced by nitrate and suppressed by ammonium in both species (**Figure 8**). Similarly, the receptor-like kinase FERONIA (LOC123440783 - functional association with lateral root development) showed stable expression under ammonium but strong induction under nitrate, particularly at higher concentrations, suggesting a conserved role in nitrate-responsive lateral root development (**Figure 8**). In contrast, PRX72 (LOC123439773 - BRADI_2G09660v3) exhibited species-specific regulation, being nitrate-induced in barley but ammonium-induced in *B. distachyon* (**Figure 8**).

**Figure 8:**
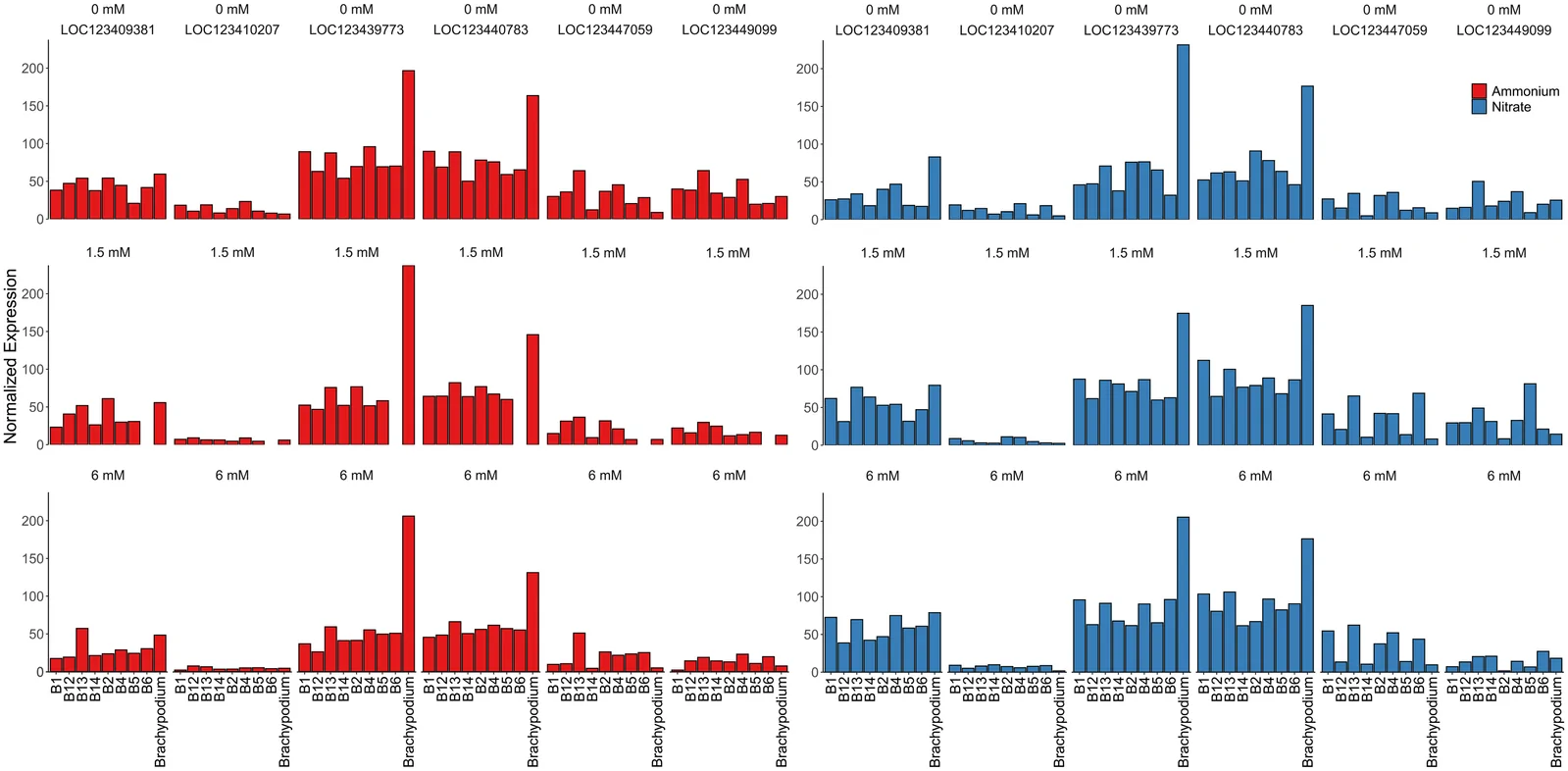
Normalized expression profiles of selected barley hub genes across barley genotypes and Bd21-3 under ammonium and nitrate treatments at three N concentrations (0, 1.5, and 6 mM). Red bars represent ammonium treatments, and blue bars represent nitrate treatments. Each panel corresponds to one gene, with bars showing normalized expression levels across genotypes and Bd21-3.

### Aspartyl protease as conserved hub genes coordinate root architecture and N responses across two species

Interestingly, the hub genes of three modules, which are correlated to the root phenotype in the Bd21–3 network BRADI_1G67440V3 (LOC123449099), BRADI_1G42930V3 (LOC123410207), and BRADI_2G35197V3 (LOC123424428) were preserved in the barley networks. In Bd21-3, BRADI_1G67440v3 (FAF-like, chloroplastic) (HORVU.MOREX.r3.4HG0390400) was negatively correlated with N content where its expression was suppressed under high N concentrations, and this effect was exacerbated in response to ammonium compared to nitrate. The homolog of this gene in barley (HORVU.MOREX.r3.4HG0390400) was present in the module green-yellow and showed a similar expression pattern in ammonium applications to Bd21-3 (**Figure 8**).

We have identified in Bd21–3 two key genes namely BRADI_1G42930v3, which encodes an aspartyl protease family protein (APs), and BRADI_1G39167v3, which encodes a phosphoenolpyruvate carboxylase 1 (PEPC 1), whose transcript levels correlated with that of hub genes which in turn correlated to root anatomical and morphological responses across varying concentrations of ammonium and nitrate. BRADI_1G42930v3 (APs) which is homologous to HORVU.MOREX.r3.7HG0674560-LOC123410207 in barley is a member of the Hv-green-yellow module. This gene was functionally characterized in *A. thaliana* and ASPR1 overexpression mutants displayed shorter primary roots and a pronounced reduction in the number of lateral roots compared to wild types (Soares et al., 2019). This gene is the first AP gene with an anticipated role in the regulation of root development in *A. thaliana* and Bd21-3 (Rouina et al., 2025; Soares et al., 2019). The expression of BRADI_1G42930v3 in Bd21–3 showed a similar pattern as most of the barley genotypes across the ammonium and nitrate application (**Figure 8**). The other hub gene in Bd21–3 mentioned above, BRADI_2G35197V3 (HORVU.MOREX.r3.1HG0019330-LOC123424428) showed an expression that was highly correlated with root cross-sectional and cortex area. However, it showed a different expression pattern in its response to N form and concentration in barley compared to Bd21-3 (**Supplementary Figure 5**).

## Discussion

### Ammonium stimulates initiation of lateral roots, suppresses primary root elongation in barley but promotes highly branched, thin roots in *B. distachyon*

In our study, the N treatments were designed to represent biologically meaningful nutritional regimes across both species. Potential confounding effects of seed N reserves in two species were minimized by growing barley and *B. distachyon* long enough before phenotyping for early seed-derived N to be largely depleted, allowing the observed root architectural responses to mainly reflect external N supply and genotype/species-specific N response strategies. This strengthens the interpretation that the contrasting RSA patterns observed under ammonium are not simply a consequence of differences in seed reserves or early developmental buffering, but rather reflect distinct species-specific responses to ammonium nutrition. Accordingly, the higher TRL and LRL for Bd21–3 in the ammonium treatments compared to barley indicate a higher tolerance to ammonium and support the notion that Bd21–3 possesses more efficient ammonium assimilation and detoxification mechanisms, a reduced sensitivity to cytosolic acidification, and more permissive developmental signaling under ammonium supply compared to barley. On the other hand, suppression of primary root elongation under ammonium observed in barley genotypes suggests that barley prioritizes stress avoidance over architectural plasticity when exposed to high ammonium availability. This adaptive trade-off is commonly associated with cytosolic acidification, interference with divalent cation uptake (Ca²^+^, Mg²^+^), and oxidative stress (Coskun et al., 2013; Shilpha et al., 2023).

Our observation for barley is in agreement with findings of Naz et al. (2021) who demonstrated that nitrate and urea supported superior root biomass and architectural traits in barley compared to ammonium and organic N sources like glycine. Nitrate enhanced root volume and tillering, while ammonium caused growth suppression and root system reduction in barley (Naz et al., 2021). Similar to Bd21-3, ammonium-tolerant RSA responses have been reported in other *Poaceae* species, including ryegrass, wheat, and rice (Belastegui-Macadam et al., 2007; Britto et al., 2001; Setien et al., 2013), and in Arabidopsis under low-to-moderate ammonium availability (Lima et al., 2010).

In barley, nitrate consistently promoted lateral root elongation, particularly at moderate concentrations (1.5 mM), whereas ammonium led to a reduction in TRL and limited LR proliferation. In contrast, *B. distachyon* (Bd21-3) displayed a distinct architectural preference for ammonium. LRL was highest under ammonium and reduced under nitrate, and although TRL declined at high ammonium concentrations, the magnitude of this decline was substantially lower than in barley.

The contrasting N responses of *B. distachyon* and barley observed here may reflect differences in ecological niche and selective pressures. As a wild temperate grass, *B. distachyon* likely evolved under heterogeneous and fluctuating soil N conditions, favoring flexible ammonium assimilation and enhanced root plasticity. In contrast, modern barley has been subjected to long-term agricultural selection in nitrate-dominated systems, potentially favoring genotypes optimized for nitrate acquisition rather than ammonium tolerance.

### Conserved nitrate-induced metabolic responses and divergent ammonium detoxification strategies

Despite the above discussed differential phenotypic reaction of barley and *B. distachyon* upon nitrate application, this triggered a consistent significant upregulation of genes associated with the PPP in both barley and Bd21-3 (**Figure 4**; **Supplementary Data Sheet 1**). In barley, PPP genes were particularly enriched in nitrate-responsive co-expression modules, especially in genotypes with higher nitrate-induced lateral root elongation. In both species, transcriptomic data show that ammonium treatment resulted in the upregulation of genes involved in glutamine biosynthesis (GS pathway) and amino acid transport, indicating a conserved metabolic route for ammonium assimilation and redistribution. While barley tends to suffer from ammonium-induced stress and shows restrained root development (**Figure 1**), Bd21–3 effectively detoxifies ammonium, remobilizes N, and activates stress-resilient ion transport systems (**Figure 1**), enabling a more plastic and adaptive RSA in response to ammonium.

#### Nitrate reduction

An increase in nitrate availability caused PPP upregulation in our experiment, which aligns with previous observations in *A. thaliana* and maize, where rapid PPP induction occurred within hours after nitrate exposure (Bussell et al., 2013; Redinbaugh and Campbell, 1998). This supports the notion that PPP activation represents a conserved mechanism among higher plants. NADPH production via PPP is essential for the functioning of nitrate reductase, which catalyzes the cytosolic reduction of nitrate to nitrite. This biochemical link is crucial because NADPH availability directly influences the capacity for nitrate assimilation (Redinbaugh and Campbell, 1998). The nitrate-induced activation of the oxidative pentose phosphate pathway in Bd21–3 indicates a conserved metabolic response that may support nitrate assimilation by supplying reducing equivalents and carbon intermediates. Nevertheless, species differed in how this assimilated N was allocated. Barley invested more strongly in lateral root elongation and branching under moderate nitrate availability, with reduced investment at high nitrate, whereas Bd21–3 likely allocated nitrate-derived resources more toward shoot development or source-related metabolism than lateral root expansion.

#### Ammonium assimilation

In Bd21-3, the upregulation of genes associated with glutamine biosynthesis indicates a potential enhancement of ammonium assimilation through the GS GOGAT pathway. This may have alleviated ammonium toxicity and supported the observed lateral root growth by maintaining nitrogen homeostasis and reducing the accumulation of free ammonium. In contrast, in barley, GS induction varied across genotypes and was lower in several of them, resulting in the accumulation of free ammonium. This genotype-dependent accumulation is known to inhibit cell elongation and contributing to primary root shortening and lower total root length (Hachiya et al., 2021; Liu et al., 2013). Studies have shown that despite high ammonium levels in the medium, internal ammonium concentrations in *B. distachyon* roots remain low, suggesting efficient detoxification (de la Peña et al., 2019). This indicates that Bd21–3 employs a specific strategy to mitigate ammonium toxicity in roots by inducing both cytosolic glutamine synthetase (GS1) and plastidic GS2 isoforms, along with downstream glutamate synthase (GOGAT) enzymes. These enzymes facilitate the production of glutamate and aspartate, two amino acids known to be involved in ammonium detoxification (de la Peña et al., 2019). Setien et al. (2013) demonstrated that insufficient GS activity in wheat under high ammonium leads to free ammonium accumulation, negatively impacting root growth due to cellular toxicity and inhibition of elongation. Within this pathway, PEP plays a crucial role (Masumoto et al., 2010), and in our study, we have identified it as a potential key regulator of RSA in Bd21-3. Glutamine also acts as a signaling molecule influencing root meristem activity and architecture (Liao et al., 2024). Its presence may also promote lateral root branching, explaining Bd21-3*’s* high-density, and fine root phenotype under ammonium. This rapid conversion of toxic ammonium into amides (mainly Gln and Asn) serves as a detoxification strategy and also a way to store and transport N efficiently (Hayashi and Chino, 1990). Consistent with this function, genes associated with the GO categories “amino acid transport,” “amino acid transmembrane transporter activity,” and “amino acid transmembrane transport” were differentially represented in both species. The enrichment analysis identified 37 unique candidates associated with amino-acid transport, including 23 in Bd21–3 and 14 in barley. In Bd21-3, amino-acid transporter-associated genes were overrepresented under both ammonium and nitrate comparisons. In barley, the response was more genotype-dependent, with transporter-associated genes detected predominantly in B1 and B13 under nitrate comparisons and in B12 and B14 under ammonium comparisons.

The ammonium detoxification cycle in plant roots is energetically expensive. In addition to the direct toxicity caused by external ammonium, the diversion of metabolic energy towards detoxification can impair overall plant growth (Lasa et al., 2002). Thus, it is possible that Bd21-3, along with fast ammonium assimilation, also has differential energy allocation strategy. The observed contrasting responses between barley and Bd21–3 to ammonium treatment could be due to differences in their carbon allocation and energy management strategies. As a wild species, Bd21–3 exhibits greater tolerance to extreme environmental conditions, such as excess ammonium by deploying a more efficient carbon investment strategy.

#### Amino acids translocation

In both barley and Bd21-3, ammonium application led to the upregulation of several amino acid transporters genes (**Figure 4**; **Supplementary Data Sheet 1**). This transcriptional activation ensures that newly synthesized amino acids can be translocated within the plant to support shoot growth, prevent accumulation in roots, and maintain cellular N balance. In Bd21-3, transporter gene induction is typically coordinated with root architectural changes, such as increased lateral root formation and higher root surface area (Krouk et al., 2010). Amino acid transporters (especially AAPs and UMAMITs) are upregulated in both species, but Bd21–3 showed a stronger coordination between transporter expression and root development (**Figure 4**; **Supplementary Data Sheet 1**). In contrast, in barley, a less efficient translocation (or higher stress burden) may lead to weaker lateral root emergence under ammonium. This dual regulation is especially critical under ammonium treatments, which can otherwise inhibit root growth, disrupt ion balance, and generate cytosolic acidification if not detoxified.

### Gene regulatory mechanisms shaping root architecture in response to N forms

The Hv-purple module, which was highly associated with LRL across both barley and Bd21-3, revealed a tightly co-expressed regulatory hub enriched for genes involved in cell wall modification, redox regulation, and developmental signaling. The core genes of this module LOC123409381 (peroxidase 55-like), LOC123440783 (FERONIA receptor kinase), and LOC123447059 (primary amine oxidase-like) are not only conserved between the two species but also transcriptionally regulated in a N form dependent manner, induced by nitrate and suppressed by ammonium. Furthermore, these genes have been functionally validated in Arabidopsis for their role in H_2_O_2_ detoxification, lignin biosynthesis, root hair growth, and root meristem activity (Fraudentali et al., 2020; Herrero et al., 2013; Li et al., 2020; L. Liu et al., 2021). These observations together illustrated the deep evolutionary integration of TF networks and environmental response mechanisms among barley and *B. distachyon*.

In barley, LOC123409381 (peroxidase 55-like) expression as a hub gene in the Hv-purple module is enhanced by nitrate supply, while ammonium supply suppressed it, a pattern partially conserved in Bd21-3. This differential regulation likely reflects the distinct cellular redox states associated with the two N sources, as nitrate assimilation requires NADPH and is closely linked to controlled redox signaling, which can promote peroxidase-mediated cell wall remodeling. In contrast, ammonium nutrition often leads to excess ROS accumulation and cellular stress, which may inhibit the expression or activity of redox-sensitive enzymes unless ROS levels are efficiently buffered. Consistent with this, nitrate availability appears to promote cell wall remodeling via redox-sensitive TF cascades (potentially involving bZIP, WRKY, and bHLH TFs), whereas ammonium-induced oxidative stress may dampen this response.

Another hub gene of the Hv-purple module LOC123447059 (CuAO-like), which is potentially under the influence of bHLH and MYB TFs, connects arginine-derived NO production to lateral root signaling via ROS generation. Its expression pattern, suppressed under ammonium in both species, implies a conserved ammonium-sensitive checkpoint regulating root proliferation. However, its higher expression under nitrate in barley again suggests a stronger nitrate-dependent activation of ROS-associated developmental cues through TF regulation. These findings reveal that while barley tends to recruit stress-buffering TFs to manage nitrate-driven elongation and ammonium-induced ROS, Bd21–3 relies more on developmentally plastic TFs to promote lateral root formation even under ammonium stress. This transcriptional divergence supports the broader hypothesis that Bd21–3 evolved a fine-tuned regulatory strategy for root proliferation under variable nutrient inputs, whereas barley’s transcriptional network prioritizes stress resilience over architectural plasticity.

LOC123440783 (FERONIA receptor kinase), another hub gene of the Hv-purple module, is co-regulated by Trihelix TFs, and previous studies suggest that it plays a critical role in root hair elongation and nutrient sensing, particularly under low N and cold stress (Pacheco et al., 2023). These findings highlight FERONIA as a key integrator of environmental and developmental signals in root adaptation. Consistent with this regulatory role, our results indicate that in barley, stress-associated TF families are more strongly linked to redox and cell wall reinforcement pathways under ammonium, whereas in Bd21-3, developmental TFs are more tightly coupled to lateral root promotion. On the other hands, enriched TFs associated with the Hv-purple module include bHLH, bZIP, WRKY, and NAC which together mediate responses to N signaling, oxidative stress, and developmental cues (Meraj et al., 2020) confirm the regulatory importance of this module in the network. These insights make the Hv-purple module and its associated TFs compelling targets for future functional validation and breeding strategies, especially to engineer improved root traits and N use efficiency in cereals through TF-guided manipulation of redox and cell wall remodeling genes. The occurrence of FERONIA-like receptor kinases and ROS-associated genes, including class III peroxidases and CuAO-like candidates, in the preserved modules further suggests a possible connection between N responses, cell-wall integrity sensing and root barrier regulation. It is known that FERONIA-related signaling can modulate ROS homeostasis and cell-wall properties, potentially coordinating root growth with changes in mineral availability. ROS-dependent cell-wall modifications may also contribute to the formation or adjustment of apoplastic barriers and thereby influence nutrient movement into the root. However, ROS production, barrier deposition and FERONIA activity were not directly measured in our study. Therefore, this proposed connection remains a mechanistic hypothesis requiring experimental validation.

Together, these observations suggest that the candidate regulators identified in this study are not species-specific in their presence but may differ in their regulatory context and contribution to N-responsive root architectural variation. The fact that related regulators have been implicated in root development or N responses across species supports the presence of conserved regulatory components. However, the contrasting RSA responses of barley and *B. distachyon* indicate that these shared components may be embedded in different regulatory hierarchies or connected to different downstream developmental outputs. Thus, the divergence between species is likely not explained by unique regulators alone, but by differences in how conserved regulatory elements are integrated into species-specific networks controlling root responses to nitrate and ammonium. This interpretation links the observed architectural divergence to differential network organization rather than to the simple presence or absence of individual genes. An important limitation of this cross-species comparison is that the species-specific networks differed in both sample size and represented genetic diversity. The barley network comprised 168 samples from eight genotypes and therefore captured genotype-dependent as well as N-dependent expression variation. In contrast, the *B. distachyon* network comprised 20 samples from the single inbred line Bd21–3 and thereby only captured treatment-dependent variation. Consequently, the barley network is expected to have greater statistical stability and broader intraspecific representation. The module-preservation results should therefore be interpreted as evidence for conserved co-expression patterns between Bd21–3 and the sampled barley diversity, rather than as a fully balanced species-wide comparison.

## Summary

This study provides a cross-species framework linking N-dependent root architectural responses with transcriptional regulation in *B. distachyon* and barley. First, ammonium and nitrate produced distinct root phenotypes, and the magnitude of these responses varied among barley genotypes. Second, transcriptomic analyses identified conserved and species-specific responses involving N assimilation, redox metabolism, cell-wall regulation and root development. Third, preserved co-expression modules prioritized candidate genes, including FERONIA-like receptor kinases, class III peroxidases, CuAO-like genes, aspartic proteases and PEPC1, that may connect N signaling with root architectural adaptation.

These findings provide candidate genes and pathways for developing cereal root systems better adapted to contrasting N forms. However, the transcriptomic patterns and predicted regulatory relationships require independent experimental validation. Translation to farming will additionally require functional testing of the prioritized genes, evaluation across broader cereal germplasm and field experiments under realistic soil and N-management conditions. Future studies should in addition determine whether these candidates can improve N acquisition and nitrogen-use efficiency without compromising crop growth or yield.

## Data Availability

RNA-seq data are available in the NCBI SRA under accession number PRJNA1348082 (Brachypodium distachyon, Bd21-3) and PRJNA1475216 (barley genotypes). The data can be accessed at the following link: https://doi.org/10.5281/zenodo.22163986.

## Supplementary material

Supplementary Data Sheet 1 Gene ontology enrichment analysis of differentially expressed genes across nitrogen-treatment comparisons in barley genotypes

Supplementary Data Sheet 2 Hub genes and transcription factors associated with root phenotypic traits in barley and Brachypodium Bd21-3 co-expression networks

Supplementary Data Sheet 3 Candidate barley hub genes, Brachypodium homologs, module assignment, and literature-supported functional annotations

Supplementary Data Sheet 4 Gene ontology enrichment analysis of module-specific genes in barley co-expression networks
