# Supplementary material for: Cross-species transcriptomic plasticity and root architectural adaptations in Brachypodium (Brachypodium distachyon) and barley (Hordeum vulgare) genotypes under different ammonium and nitrate availability
Source: Front Plant Sci. 2026 Oct 9;17:1916213. doi: 10.3389/fpls.2026.1916213 (PMC13690746; doi:10.3389/fpls.2026.1916213)
Supplement: Supplementary file 1 [file Supplementaryfile1.docx]

**Supplementary fig-1:** Experimental design for phenotyping and transcriptome analysis of *Brachypodium distachyon* and barley under nitrate and ammonium nutrition.

**B**

**A**

**A**

**C**

**D**

**Supplementary fig-2:** Genotypic variation in root traits of barley under ammonium and nitrate treatments. (A) TRL across N concentration and form (ammonium red, nitrate blue) for eight barley genotypes. (B) LRL for the same genotypes and treatments. (C) Partitioning of total biomass between shoots (blue) and roots (red) for barley genotypes under ammonium and nitrate treatments. (D) Representative root system images of barley genotypes grown under 1.5 mM ammonium (top panel) and nitrate (bottom panel). Error bars represent standard deviations, and different letters indicate statistically significant differences among genotypes within each nitrogen source (Tukey’s HSD, p < 0.05).

**Ammonium**

**Nitrate**

**Supplementary fig-3:** Differentially expressed genes (DEGs) in barley genotypes and Brachypodium under nitrate (left) and ammonium (right) treatments. Each panel shows the number of upregulated (blue) and downregulated (red) genes in pairwise comparisons of N concentrations: Low vs Moderate, Low vs High, and Moderate vs High. Counts are displayed for each genotype and N source separately. Bar length represents DEG counts, and numbers indicate the exact counts per category.

**Supplementary fig-4:** Module-trait relationships identified by WGCNA in barley. Each cell represents the correlation coefficient (top) and p-value (in parentheses) between a module eigengene (rows) and a measured phenotypic trait (columns). The color scale indicates the strength and direction of the correlation, with green representing negative correlations and red representing positive correlations.

**Supplementary fig-5:** Expression patterns of the BRADI_2G35197V3 (serine carboxypeptidase 2) under ammonium and nitrate treatments. (Top) Normalized expression of BRADI_2G35197V3 in Brachypodium across three N concentrations (0, 1.5, and 6 mM) for ammonium (red) and nitrate (blue). (Bottom) Genotype-specific normalized expression of the barley homolog LOC123424428 in the same N treatments.

**Supplementary fig-6:** Principal component analysis of barley RNA-seq data before and after batch correction.

**Supplementary Table 1:** ANOVA results of total (first) and lateral root length (second) in Bd21-3. Effects of N concentration, N source, block, and the N concentration × N source interaction on total root length (TRL) and lateral root length (LRL) of Bd21-3. The analysis included 0, 1.5, and 6 mM Nitrogen.

|  | Df | SumSq | MeanSq | F | value | Pr(>F) |
| --- | --- | --- | --- | --- | --- | --- |
| ncon | 2 | 4460236 | 2230118 | 22.971 | 2.72E-05 | *** |
| nsou | 1 | 1825329 | 1825329 | 18.801 | 0.000588 | *** |
| block | 3 | 812522 | 270841 | 2.79 | 0.07656 | . |
| ncon:nsou | 2 | 35100 | 17550 | 0.181 | 0.836417 |  |
| Residuals | 15 | 1456266 | 97084 |  |  |  |

|  | Df | SumSq | MeanSq | F | value | Pr(>F) |
| --- | --- | --- | --- | --- | --- | --- |
| ncon | 2 | 3027147 | 1513574 | 20.65 | 4.92E-05 | *** |
| nsou | 1 | 1247678 | 1247678 | 17.023 | 0.000898 | *** |
| block | 3 | 559788 | 186596 | 2.546 | 0.095076 | . |
| ncon:nsou | 2 | 12853 | 6426 | 0.088 | 0.916523 |  |
| Residuals | 15 | 1099426 | 73295 |  |  |  |

Significance codes: *** P < 0.001; ** P < 0.01; * P < 0.05; . P < 0.10; ns, not significant.
